# Items

**Published:** 1898-03

**Authors:** 


### Items.

---

ANNOUNCEMENT.—In the April issue of the JOURNAL the publication of the History of Medicine in the County of Erie will begin. This will be a succinct account of medical affairs in the county during the nineteenth century. It will deal with men of prominence in the early history of the county, with medical societies, medical colleges, hospitals, medical journals, medical officers in the civil war, women physicians, history of homeopathy, individual members of the profession and many other items of interest. It will be profusely illustrated and will be published as a serial occupying a year in its publication. It forms the medical chapter in Our County and Its People, written by William Warren Potter, M. D., and is republished in the JOURNAL by courtesy of the Boston History Company.

The volume begins in August. Subscribers remitting now will receive the JOURNAL free until that date. Subscription price, $2.00 a year in advance.

---

AN ARMY MEDICAL BOARD will be in session at Washington City, D. C., during the month of May for the examination of candidates for appointment to the medical corps of the United States army to fill existing vacancies. Persons desiring to present themselves for examination by the board will make application to the Secretary of War before April 15, 1898, for the necessary invitation, giving the date and place of birth, the place and state of permanent residence, the fact of American citizenship, the name of the medical college from which they were graduated and a record of service in hospital, if any, from the authorities thereof. The application should be accompanied by certificates based on personal acquaintance, from at least two reputable persons, as to his citizenship, character and habits. The candidate must be between 22 and 29 years of age and a graduate from a regular medical college, as evidence of which his diploma must be submitted to the board. Successful candidates at the coming examination will be given a course of instruction at the next session of the Army Medical School, beginning in November, 1898.

Further information regarding the examinations may be obtained by addressing the Surgeon-General, U. S. Army, Washington, D. C.

---

The Medical Excursion in June to Denver and Salt Lake City.—The American Medical Association meets at Denver, June 7th to 10th. One of the features of the gathering will be an excursion from Denver to Salt Lake City and return via the D. & R. G., Colorado Midland and Rio Grande Western railways, through the "heart of the Rockies," furnishing a splendid opportunity to view the most magnificent scenery on the American continent. Salt Lake City is an ideal summer resort and the bathing at Saltair in the Great Salt Lake—inland salt sea nearly a mile above sea level—is superb in June. There are more attractions in and about Salt Lake City than any place in the world. Later notice will appear in the Journal giving rates for this excursion and all details. In the meantime send to F. A. Wadleigh, G. P. A., Rio Grande Western Railway, Salt Lake City, for copy of pamphlets on Salt Lake City and the Rocky Mountains.

---

Orthoform should be obtained by the physician from his pharmacist for about $2.00 per oz. Should any practitioner who desires to employ the remedy be unable to readily obtain it in his vicinity, Victor Koechl & Co., 79 Murray St., New York, will be glad to fill his order direct upon receipt of the above mentioned amount. As yet only Orthoform (the base) is obtainable.

---

The Medical Society of the County of Erie, at its last meeting, authorised the board of censors to employ legal assistance in enforcing the laws against illegal practice of medicine. We understand the board has engaged the services of Tracy C. Becker, Esq., and Mr. Marc W. Comstock—the first named as counsel and the latter as attorney.

